# Beneficial effects of Apelin-13 on metabolic diseases and exercise

**DOI:** 10.3389/fendo.2023.1285788

**Published:** 2023-11-28

**Authors:** Ruiming Wen, Ruiqi Huang, Ke Xu, Yang Cheng, Xuejie Yi

**Affiliations:** ^1^ School of Sports Health, Shenyang Sport University, Shenyang, Liaoning, China; ^2^ School of Physical Education, Liaoning Normal University, Dalian, Liaoning, China

**Keywords:** adipokine, Apelin-13, adipose tissue, metabolic disease, exercise

## Abstract

Apelin, a novel endogenous ligand of the G-protein-coupled receptor APJ, is encoded by the *APLN* gene and can be hydrolyzed into multiple subtypes, with Apelin-13 being one of the most active subtypes of the Apelin family. Recent studies have revealed that Apelin-13 functions as an adipokine that participates in the regulation of different biological processes, such as oxidative stress, inflammation, apoptosis, and energy metabolism, thereby playing an important role in the prevention and treatment of various metabolic diseases. However, the results of recent studies on the association between Apelin-13 and various metabolic states remain controversial. Furthermore, Apelin-13 is regulated or influenced by various forms of exercise and could therefore be categorized as a new type of exercise-sensitive factor that attenuates metabolic diseases. Thus, in this review, our purpose was to focus on the relationship between Apelin-13 and related metabolic diseases and the regulation of response movements, with particular reference to the establishment of a theoretical basis for improving and treating metabolic diseases.

## 1 Introduction

The increasing prevalence of metabolic diseases, especially those associated with a sharp increase in the incidence of obesity-related diseases, poses a major threat to human health worldwide (1). These diseases, which are mainly caused by local or systemic metabolic abnormalities, due to an imbalance in energy metabolism (2), include diabetes mellitus (DM), obesity, and metabolic bone disease, among others (3, 4). Such metabolic disorders are accompanied by the abnormal secretion and/or dysfunction of many cytokines (5).

Apelin is mainly synthesized and secreted by white adipocytes (6), and is expressed in metabolic tissues, such as cardiovascular, skeletal, renal, etc (7, 8), while its expression in white adipose tissue (WAT) is higher than that in liver, muscle and brown adipose tissue(BAT), and with the differentiation of adipocytes, the expression of Apelin also increases significantly. Therefore, it is called “adipokine” (6, 9, 10). Apelin-13 is considered the most functional subtype of the Apelin family, and it actively participates in regulating bone marrow mesenchymal stem cell (BMSC) apoptosis, osteoblast differentiation, glucose and lipid metabolism, and other physiological processes (11–13), which means that it has an important role in the prevention and treatment of metabolic diseases such as obesity, diabetes and osteoporosis; however, there is still some dispute regarding its regulatory effect on metabolic diseases (14, 15). Therefore, it is extremely important to explore the physiological mechanisms through which Apelin-13 affects various metabolic diseases.

In addition, Apelin-13 can also serve as a “motion-sensitive factor” and can be used to increase the potential of exercise (16). Long-term exercise can also promote the expression of Apelin-13 in the serum of obese individuals (17), suggesting that exercise might regulate metabolic diseases through Apelin-13. Therefore, this article focuses on the role of Apelin-13 in metabolism and its potential relationship with exercise, to provide new ideas for improving metabolic diseases through exercise.

## 2 Overview

### 2.1 Apelin-13 structure

In 1998, Tatemoto et al. extracted a peptide called Apelin from bovine gastric secretions (18), and this was found to be translated from the *APLN* gene located on chromosomes Xq25-26.1. Subsequent studies showed that *APLN* in humans, mice, and cattle have high structural homology (19). Apelin has also been confirmed to be an endogenous ligand of the G-protein-coupled receptors APJ and Elabela (ELA) (20, 21); moreover, it activates the receptor signaling pathway by binding to APJ and performs specific biological functions (21). Apelin can be hydrolyzed into subtypes of different lengths (Apelin-12, Apelin-13, Apelin-17, Apelin-36, etc.) (22). The N-terminal residue of pro-Apelin can be translated, modified by endogenous peptidases, and cleaved with high catalytic efficiency to remove phenylalanine at the C-terminus to form Apelin-36, which is then cleaved in a similar manner into smaller Apelin-17 and Apelin-13 fragments (23–25). These subtypes always retain their significantly biologically active C-terminus during this process (26, 27). Moreover, the endogenous peptidases that have a predominant role in this process typically include angiotensin converting enzyme 2 (ACE2) and neutral endopeptidases, among others (28). In addition, these subtypes can all bind APJ receptors, but different conformational states of their receptors might affect protein activity (29). The activity of each subtype is different, and shorter subtypes can more effectively activate the APJ receptor. Accordingly, the activity of Apelin-13 and Apelin-17 is much stronger than that of Apelin-36, and the different binding affinities for their receptor determine the different APJ signaling pathways activated in cells (30, 31). Therefore, Apelin-13 is considered to be the most biologically active affinity fragment among Apelin subtypes (32), and related research on this form is relatively extensive.

### 2.2 Biological functions of Apelin-13

#### 2.2.1 Apelin-13 affects food intake by regulating the central nervous system

The active substances secreted by adipose tissue can pass through the blood–brain barrier and are released into the central system to exert their effects and regulate energy homeostasis by integrating the internal environment and cellular signals to generate behavioral responses that can initiate or terminate feeding (33–35). One study indicated that the chronic intracerebroventricular (third ventricle) injection of Apelin-13 (1 μg, administered via slow infusion over 24 h for 10 consecutive days) significantly increased the intake of food and water, as well as the body temperature and body weight of wild-type mice (36). However, another study found that the acute intracerebroventricular (2 μg, injection within 15 min) injection of Apelin-13 significantly reduced the intake of food and water, as well as the rate of respiratory exchange in C57BL/6 rats (37); the discrepancy between these two studies was mainly attributed to inconsistencies in the injection speed. Interestingly, the intracerebroventricular injection of Apelin-13 did not exert a significant effect on the food intake and body weight in rats fed a high-fat diet (37). In their attempts to determine possible reasons for this, the authors found that the mRNA expression of APJ in the hypothalamus, which had been significantly increased in the high-fat diet group, was suppressed by the injection of Apelin-13, whereas this was not observed in rats fed a normal diet (37). This indicated that APJ might be involved in the regulation of diet and also that APJ could be affected by different physiological and pathological states. Conversely, the acute intracerebroventricular injection of the receptor APJ-specific antagonist Apelin-13 (F13A) and the corticotropin-releasing factor (CRF) receptor antagonist α-helical CRF9-41 had no significant effect on food intake in Kunming mice (Derived from Swiss mice) but significantly alleviated the reduction in food intake caused by the acute injection of Apelin-13 (38). This indicated that Apelin-13 might be involved in activating central APJ receptors, as well as CRF receptors, thereby directly affecting the activity of the hypothalamus–pituitary–adrenal axis, which reduces food intake, which may be affected by different genetic backgrounds. The above report indicates that Apelin-13 can stimulate the central nervous system, thereby affecting food intake, and may be involved in maintaining energy balance in the body under physiological conditions.

#### 2.2.2 Apelin-13 participates in regulating the inflammatory response

Numerous studies have shown that Apelin-13 not only participates in the central regulation of food intake but also exerts anti-inflammatory effects in various pathological states (39, 40). After subarachnoid hemorrhage, the protein expression of Apelin-13 and APJ in the left basal cortex of the brain is significantly increased. Moreover, the exogenous injection of Apelin-13 was found to significantly reduce thioredoxin interaction protein (TXNIP), nucleotide binding oligomeric domain like receptor protein 3 (NLRP3), interleukin-1β (IL-1β), and tumor necrosis factor-α (TNF-α) levels. In addition, the suppression of APJ actually eliminates the protective effects of Apelin-13 on the brain with respect to myeloperoxidase and reactive oxygen species production (41). Another study found that Apelin-13 can also reduce IL-1β and TNF-α levels in the hippocampus of rats with streptozotocin-induced Alzheimer’s disease. Further, the expression of β-lactamase reduces hippocampal cell loss, thereby alleviating cognitive impairment (42). As the degree of human intervertebral disc degeneration worsens, the expression of APJ in the nucleus pulposus tissue (NP) gradually decreases. Recombinant Apelin-13 activates the APJ receptor of nucleus NP and increases the expression of type II collagen, aggrecan, sex determining region Y-box protein 9 (SOX9), matrix metalloproteinase-3 (MMP-3), and MMP-13, in addition to reducing the expression of IL-6 and TNF- α in NP cells. Moreover, the use of Ala13 (an inhibitor of Apelin-13) can reduce the expression of phosphatidylinositol 3-kinase (PI3K) and protein kinase B (AKT), whereas LY294002 can diminish the effect of Apelin-13 on NP cells (43). This indicates that Apelin-13 can inhibit the inflammatory response through the PI3K/Akt signaling pathway, thereby improving intervertebral disc degeneration. These results demonstrate the anti-inflammatory effect of Apelin-13, indicating its role as a “protective factor”, but most studies are limited to its associated phenotype and have not delved into the underlying molecular mechanisms.

#### 2.2.3 Role of Apelin-13 in autophagy and cell apoptosis

Autophagy is a dynamic recycling system for intracellular degradation, through which intracellular substances are transported to lysosomes and degraded, thereby accelerating cell renewal (44). A clinical data showed that mRNA and protein expression of Apelin in NP tissues of patients with intervertebral disc herniation were significantly lower than those of the control group, suggesting that Apelin-13 may play a role in disc herniation. Subsequently, the author found that in the H_2_O_2_ induced oxidative stress model of NP cells, Apelin-13 treatment increased mRNA and protein expression of collagen II and polysaccharides in NP cells, and increased autophagy flux (LC3II/I increased and p62 decreased) (45), indicating that Apelin-13 may be a beneficial factor in preventing and treating intervertebral disc herniation. Similarly, the injection of Apelin-13 into the right ventricle significantly increases Bcl-2/Bax to alleviate neuronal apoptosis in mice with cerebral ischemia/reperfusion. This treatment also inhibits the expression of LC3B in the hippocampus and upregulates p62 expression. Acridine orange staining showed that Apelin-13 treatment significantly inhibits the increase of autophagic vacuoles (46), suggesting that it can prevent ischemic stroke injury by inhibiting excessive autophagy and cell apoptosis. Further, Wang et al. found that Apelin-13 treatment significantly reduces the mRNA expression of malondialdehyde (MDA), superoxide, and nitrotyrosine in the myocardium of mice with cardiac ischemia/reperfusion and reduces the contents of redox indicators (cytoplasmic lactate/the pyruvate ratio). However, the use of PI3K inhibitors actually eliminates this effect (47). Therefore, the targeted regulation of Apelin-13 inhibits autophagy and cell apoptosis, providing new insights into the modulation of this protein to improve body homeostasis. It is worth noting that the relevant molecular mechanism of apelin-13 improving different types of diseases through autophagy remains to be studied, which has a strong guiding significance for the prevention and treatment of metabolic diseases.

## 3 Role of Apelin-13 in metabolic diseases

### 3.1 Apelin-13 participates in the regulation of obesity

Obesity, a chronic and progressive process that affects the energy balance in the body, not only causes the abnormal or excessive accumulation of body fat (48) but also induces inflammation and fibrosis of the WAT, leading to local or systemic metabolic dysfunction (49). Aggravation linked to obesity is closely related to the release of bioactive adipokines (50). Adipokines released into the circulation act as classic hormones affecting the tissue and organ metabolism via specific receptors on the surfaces of target cells (51), thereby regulating energy metabolism (52), as well as immune system activities (53).

#### 3.1.1 Apelin-13 inhibits obesity

A large amount of data indicates that, obesity usually results in subcutaneous and visceral fat dilation (54). As one of the components of visceral fat, cardiac epicardial fat (EAT) is mainly composed of WAT, and dietary and exercise interventions can partially promote its browning (55, 56). The thickness of the EAT in obese patients is significantly higher than that in normal individuals. Moreover, the expression of Apelin in the EAT is 2.6 times higher than that in subcutaneous adipose tissue, and it is positively correlated with left ventricular diastolic function (57). This suggests that Apelin-13 might play a role in EAT and could serve as a new target for the prevention and treatment (55). Animal studies have found that amidation-modified Apelin-13 might significantly reduce the average diameter of adipocytes, mRNA and protein expression of peroxisome proliferator-activated receptor γ (PPARγ) levels and perilipin 1 (PLIN1), thereby improving the dyslipidemia caused by obesity (14). Cell-based experiments have also shown a similar trend and found that Apelin-13 reduced lipid content and total cholesterol content (14), indicating that amidation-modified Apelin-13 might downregulate the expression of PPARγ, thereby inhibiting the differentiation of adipocytes, resulting in the regulation of PLIN1 expression to promote lipolysis *in vivo*, which ameliorated obesity symptoms. Reducing lipid storage in the body is another important mechanism that reduces obesity (58). Aquaporin 7 (AQP7), a transporter protein present in the plasma membrane of adipocytes, is positively correlated with the degree of obesity (59). AQP7 deficiency in adipocytes increases glycerol kinase activity and accelerates triglyceride synthesis, ultimately leading to obesity (60). *In vitro* experiments have confirmed that Apelin-13 significantly upregulates the expression of AQP7 in palmitic acid-treated hypertrophic adipocytes and reduces the accumulation of triglycerides in the cytoplasm, which could be reversed using the PI3K inhibitor LY294002 (61), suggesting that Apelin-13 might suppress lipid storage in hypertrophic adipocytes and thereby reduce obesity by upregulating AQP7 expression via the PI3K signaling pathway. These findings indicate that Apelin-13 can inhibit adipocyte differentiation, promote fat breakdown, and reduce lipid storage, thereby alleviating the pathological changes caused by obesity.

Apoptosis is a highly regulated form of cell death that enables specific cells to be sacrificed for the greater good of the organism (62, 63). An increase in the volume of adipose tissues, which causes obesity, could be due to an increase in the number and/or size of adipocytes (64). Moreover, an increase in adipocyte apoptosis might prevent the excessive accumulation of adipose tissue, thereby ensuring the stability of the physical condition in the body (65). Altered adipokine production could affect the balance between apoptosis and survival, ultimately determining cell fates. According to one study, the activity and quantity of adipocytes in obese rats are significantly higher than those in a normal control group (66). Further, following the tail vein injection of amidation-modified Apelin-13 into obese rats, the serum levels of blood glucose, free fatty acids, triglycerides, total cholesterol, and low-density lipoprotein cholesterol, as well as the expression of B-cell lymphoma-2 (BCL-2) in adipose tissues, decrease, whereas those of caspase-3 increase (67). In addition, intervention using amidation-modified Apelin-13 also diminishes the activity of 3T3-L1 preadipocytes and enhances cell apoptosis (67), indicating that amidation-modified Apelin-13 might alleviate obesity by promoting adipocyte apoptosis.

#### 3.1.2 Apelin-13 accelerates obesity

The conclusion found by Suat et al. (15) is contrary to the above results (61, 67). Apelin-13 can increase serum levels of total cholesterol and low-density lipoprotein cholesterol in a dose-dependent manner, thereby accelerating lipid metabolism disorders and significantly reducing mRNA expression levels of uncoupling protein 1 in white adipose tissue and brown adipose tissue at the scapula, as well as uncoupling protein 3 in the biceps brachii muscle (15), The increase in uncoupling protein 3 content is positively correlated with the mitochondrial content of skeletal muscle (68), indicating that Apelin-13 may reduce adipose tissue browning, mitochondrial content of skeletal muscle, and energy consumption, thereby exacerbating obesity. The report also pointed out that Apelin-13 can increase the food intake and body weight of mice (15), and this difference may be attributed to changes in their eating behavior and pathological state.

**Figure 1:**
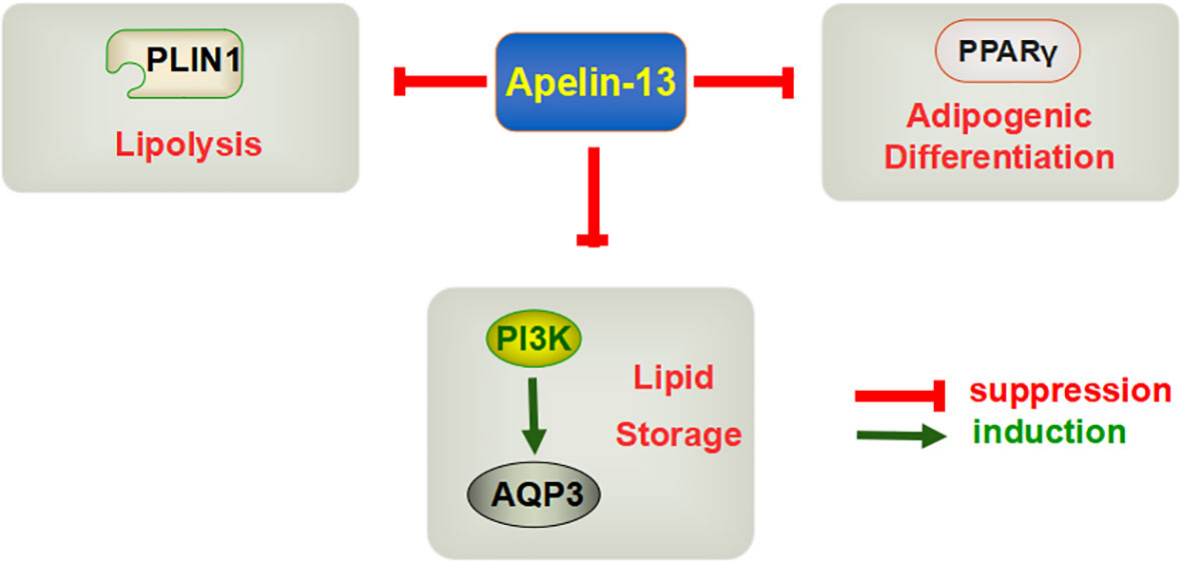
Mechanism through which Apelin-13 regulates obesity.

Generally, Apelin-13 mainly improves obesity by inhibiting adipocyte differentiation、 promoting fat decomposition and cell apoptosis (**Figure 1**). However, there are also different sounds, that is, exogenous injection of apelin-13 can also reduce energy consumption in mice, but due to the lack of existing reference basis, it still needs further verification. At present, the effect of the central and circulatory injection of Apelin-13 on different pathological conditions remains an important issue that requires a resolution. Studies focusing on the regulatory effects of Apelin-13 on obesity are relatively limited, and the molecular mechanisms underlying this process require further exploration.

### 3.2 Apelin-13 participates in regulating diabetes

Epidemiological data indicate that DM, one of the most common and increasingly prevalent metabolic diseases worldwide, could affect 693 million adults by 2045 (69). According to its clinical characteristics, DM can be divided into four types, as follows: type 1 diabetes mellitus (T1DM); type 2 diabetes mellitus (T2DM); gestational diabetes mellitus (GDM); and specific types of DM with other causes (70). Although researchers have made great progress in understanding and treating DM, the morbidity and mortality caused by this disease and related complications continue to increase (71, 72). Therefore, effective and simple biomarkers that enable the early diagnosis, progress monitoring, and targeted treatment of DM are urgently needed.

#### 3.2.1 Apelin-13 and T2DM

Research has found that the intraperitoneal injection of Apelin-13 (200 μg/kg/day, 4 weeks) can enhance the expression of Apelin-12, glucose transporter 4 (GLUT4), and AMP-activated protein kinase (AMPK) α-2 in the serum, as well as in the myocardial and aortic tissues of rats with DM. I can also improve cardiac functions (decreased heart rate and left ventricular end systolic pressure, and increased maximum rise/fall rate of the left ventricular pressure), insulin resistance, endothelial function (decreased endothelin-1 (ET-1) and increased NO and constitutive nitric oxide synthase (cNOS) activities), and the inflammatory response in rats with DM (73). Sabry et al., reported that the subcutaneous injection of Apelin-13 (0.1 mol/kg/day, 6 weeks) reduces the mRNA expression of angiotensin II (Ang II)-type 1 receptor (ATR1) in the adipose tissue of rats with T2DM and increases the levels of angiotensin-converting enzyme 2 (ACE2) and NO. The application of L-NAME, an inhibitor of cNOS, reverses the significant effects exerted by Apelin-13 (74). ACE2 converts Ang II to Ang1-7 or Ang I to Ang1-9, thereby counteracting the effects of the renin–angiotensin system on the cardiovascular system (75), suggesting that the beneficial effects of Apelin-13 on glucose and lipid metabolism in rats with DM are mainly mediated by the NO activation pathway and/or ACE1/Ang (1-7). The intraperitoneal injection of Apelin-13 (0.1 µmol/kg/day,10 weeks) decreases the glycogen content and increases the mRNA expression of GLUT4 in the myocardium, while increasing the insulin (INS) content in serum and improving the function of pancreatic β cells (76). Apelin-13 affects pathological changes in T2DM by modulating glucose metabolism. In addition, Apelin-13 decreases the fatty acid (FA) content in myocardial tissue (76). The FA content in myocardial tissue mainly depends on the uptake, oxidation, and utilization of glucose (77, 78). This function can be realized by activating PPAR-α in the heart (79, 80). Researchers have further found that Apelin-13 might also reduce the mRNA expression of FA transporters, cluster of differentiation 36 (CD36), carnitine palmitoyl transferase-1 (CPT-1), and PPAR-α in myocardial tissue (76), indicating that the mechanism underlying the regulatory effects of Apelin-13 on FAs might occur via PPAR-α inhibition, which affects lipid metabolism, thus reducing myocardial FA uptake and oxidation. Altered mitochondrial functions comprise another process through which the utilization of cardiac FAs is increased (81). To explore the effect of Apelin-13 on the biogenesis of myocardial mitochondria in rats with T2DM, Feng et al. detected the expression of peroxisome proliferator-activated receptor-γ coactivator 1α (PGC1-α) and citrate synthase in the myocardium and found that Apelin-13 did not affect the expression of either (76), suggesting that Apelin-13 might not affect the biogenesis of myocardial mitochondria in rats with T2DM. Notably, they also reported that Apelin-13 might not cause significant changes in serum FAs and blood lipid indicators (76), and this conclusion contradicted the results of previous studies (67, 73). This observation could be attributed to the minimal effect of Apelin-13 on basal fat decomposition *in vivo* or resistance to Apelin (82), which implies that the association between Apelin-13 and lipid metabolism needs to be further elucidated. These findings confirmed that Apelin-13 might attenuate the pathological changes caused by T2DM by regulating multiple signaling pathways, and thus, this has potential as a new clinical marker.

#### 3.2.2 Apelin-13 and T1DM

Similarly, Apelin-13 exerts a similar protective effect on T1DM and GDM. The intravenous injection of Apelin-13 (400 pmol/kg twice per day for 10 weeks) significantly improves islet mass and INS levels in Akita mice with spontaneous T1DM Akita and inhibits the expression of inositol-requiring protein 1α (IRE1α) and phosphorylation of protein kinase R (PKR)-like endoplasmic reticulum kinase (PERK), two endoplasmic reticulum (ER) stress receptors in pancreatic tissues. Thus, the inhibition of c-Jun N-terminal kinase (JNK) phosphorylation and expression of the pro-apoptotic transcription factor C/EBP homologous protein (CHOP) (83) indicates that Apelin-13 might inhibit ER stress-dependent cell death in the pancreas of Akita mice to some extent. Next, the authors explored the mechanism through which Apelin-13 regulates of ER stress and found that it increases the phosphorylation levels of extracellular regulated protein kinases (ERK), protein kinase B (Akt), and AMPK (83), suggesting that Apelin-13 might widely regulate multiple pathways, thereby improving ER stress caused by T1DM.

#### 3.2.3 Apelin-13 and GDM

Another study found that the subcutaneous injection of Apelin-13 (2 mg/kg) decreases serum superoxide dismutase (SOD) and glutathione peroxidase (GPx) expression in mice with GDM, which was found to significantly improve glucose and lipid metabolism disorders, oxidative stress, and inflammatory responses. Subsequently, the authors found that the phosphorylation levels of PI3K and Akt in the placenta of mice with GDM had increased significantly following the injection of Apelin-13. Moreover, the injection of LY294002, a PI3K inhibitor, reversed this process (84), suggesting that Apelin-13 might improve glucose and lipid metabolism, reduce oxidative stress and inflammation, and improve GDM by activating the PI3K/Akt signaling pathway. Human studies have shown that the serum levels of Apelin-13, Apelin-36, and NO are higher in patients with GDM than in healthy mothers in the second trimester of pregnancy, who suffer from glucose and lipid metabolism disorders (85). However, the causal relationship between abnormal Apelin-13 expression and health is worthy of further investigation.

**Figure 2:**
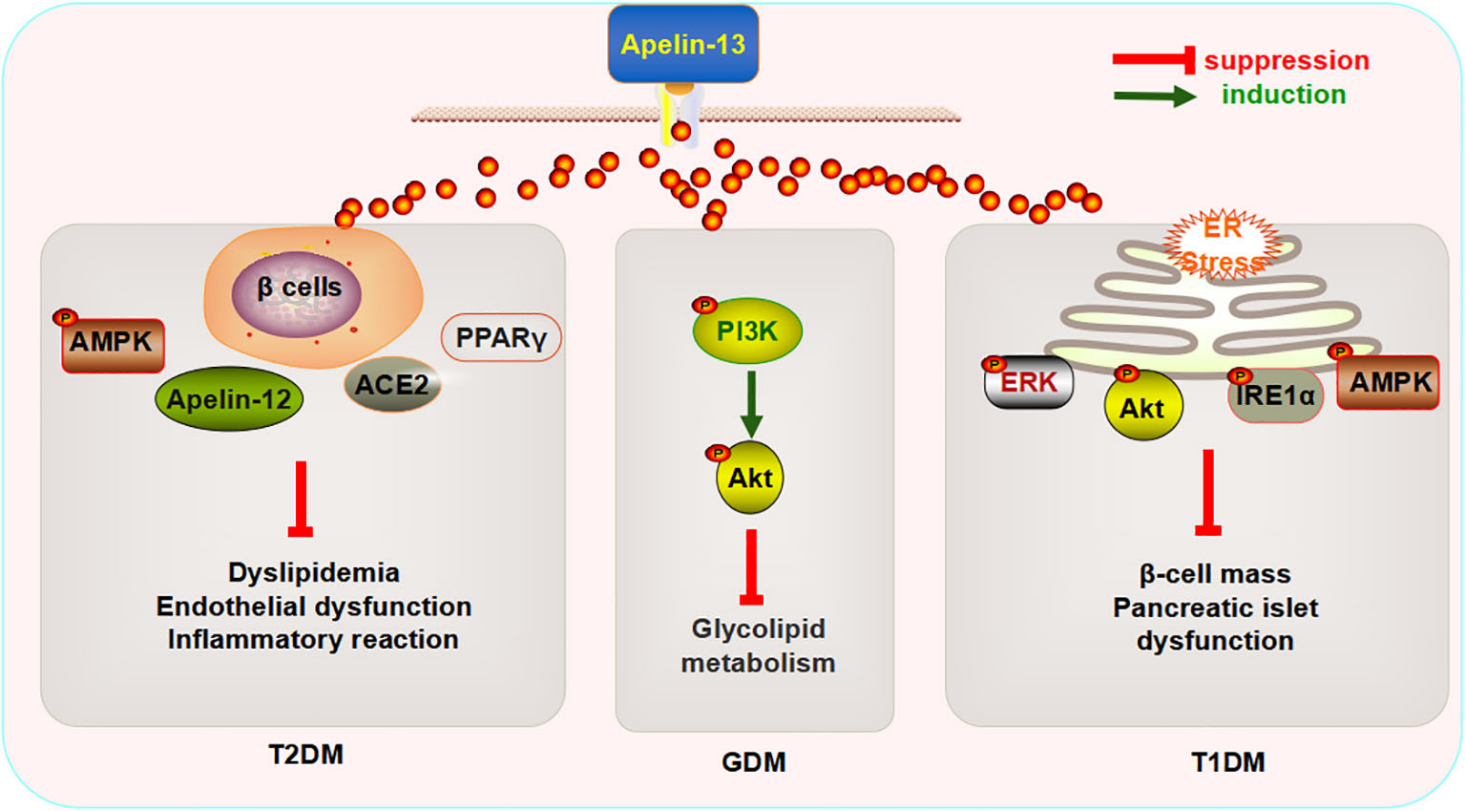
Mechanism underlying the regulatory effects of Apelin-13 on diabetes mellitus.

The aforementioned data indicate that the exogenous injection of Apelin-13 can improve glucose and lipid metabolism disorders, endothelial dysfunction, inflammatory reactions, and pancreatic islet functions in the context of diabetes by regulating multiple signaling pathways, β-cell quality, ER stress, and oxidative stress, and this protein could therefore become a biomarker for the prevention and treatment of metabolic diseases such as DM (**Figure 2**).

### 3.3 Apelin-13 is involved in the regulation of metabolic bone disease

Osteoporosis is characterized by uncoupled bone resorption, resulting in low bone mass, microstructural damage, and structural deterioration, thereby increasing the possibility of a fracture (86). In some individuals, osteoporosis is caused by chronic hyperglycemia, advanced glycation end products, and oxidative stress (87). One study found that the serum levels of Apelin-13 and 25-hydroxyvitamin D3 (25(OH)D3) are significantly lower in patients with osteoporosis than in healthy people (88), suggesting that Apelin-13 might be associated with bone metabolism. Moreover, a clinical trial based on T2DM patients found that the levels of Apelin-13, bone formation sensitive factor procollagen type I N-propeptide (PINP), and bone mineral density (BMD) in the serum of T2DM patients with osteoporosis were significantly lower than those in T2DM patients with normal bone mass, whereas the bone resorption marker type I collagen carboxyterminal telopeptide (ICTP) showed the opposite trend (89). A correlation analysis has also shown that Apelin-13 levels are positively correlated with BMD and PINP levels and negatively correlated with ICTP levels (89). This suggests that Apelin-13 might be useful as a biomarker for monitoring bone metabolism, although the mechanism underlying the interaction between Apelin-13 and bone formation or bone resorption remains unclear.

The increase in bone mass mediated by adipokines is a multifactorial process, involving various pathways that regulate the osteogenic and adipogenic differentiation of BMSCs and bone resorption (90). A large volume of data indicates that multiple signaling pathways, such as Wnt/beta-catenin (β-catenin) and PI3K/Akt, might be directly involved in the regulation of bone metabolic processes (91, 92). Studies have also shown that the total bone marrow cavity area, periosteum, and intracortical bone surfaces are decreased in Apelin-13-deficient mice, whereas the expression levels of collagen type III alpha 1 (Col3α1) and collagen maturation associated genes, namely *loxl3* and *loxl4*, are significantly downregulated (88), suggesting that Apelin-13 might play an important role in bone homeostasis. *In vitro* experiments revealed that the expression levels of Wnt-target factors, specifically Axin2, CyclinD1, Smad6, and Wisp2, as well as downstream β-catenin, are significantly downregulated in primary bone cells isolated from Apelin-13^−/−^ mice (88), indicating that Apelin-13 could be involved in regulating signaling via the canonical Wnt pathway to prevent osteoporosis. Similarly, the exogenous injection of Apelin-13 was found to reduce the gap distance of cortical bone defects in a rat tibial osteotomy model and increase the number and thickness of trabeculae to accelerate bone healing. Further, *in vitro* studies found that the administration of recombinant Apelin-13 to human BMSCs upregulates expression levels of the osteogenesis-related gene *Col1α1* and the runt-related transcription factor 2 (*Runx2*) (12). In terms of the underlying mechanism, Apelin-13 might upregulate the levels and activity of total β-catenin in cells, which is then eliminated when an appropriate concentration of DKK1, an effective inhibitor of Wnt/β-catenin signaling, is reached. Similar to that with its exogenous expression, the endogenous overexpression of Apelin-13 via a lentiviral vector also increases the expression levels of osteogenesis-related genes, and alkaline phosphatase (ALP) and alizarin red S staining showed that calcium deposition is accelerated under these conditions (12). This suggested that Apelin-13 might promote the osteogenic differentiation of BMSCs via the Wnt/β-catenin signaling pathway.

Mitochondrial autophagy is the main cellular stress response system that damages organelles and protein immunity, and it plays a key role in the differentiation, apoptosis, and survival of BMSCs (93). Liang et al. found that compared with that in wild-type rats, the expression of Apelin-13 and its receptor APJ is significantly downregulated in the distal femur of ovariectomized rats with osteoporosis. Moreover, after H_2_O_2_ treatment, PINK1, Parkin, Bax, and cytochrome C (Cyt-C) are increased, which is accompanied by a decrease in the expression of the anti-apoptotic protein Bcl-2. The exogenous injection of Apelin-13 promotes the activation of mitochondrial autophagy proteins (LC3-II, PINK1, and Parkin), thereby alleviating oxidative stress, mitochondrial dysfunction, and apoptosis in BMSCs, restoring bone mass and microstructure, increasing levels of the osteogenic genes *RUNX2*, *COL1α1*, and OCN, and downregulating expression of lipid marker PPARγ. *In vitro* experiments also showed that *AMPK-α* gene knockout partially suppresses the role of Apelin-13 in mitotic activation and anti-apoptotic mechanisms (94), suggesting that the AMPK pathway serves as an important pathway through which Apelin-13 promotes BMSC proliferation and osteogenic differentiation. This also suggests that Apelin-13 could have an important role in the adipogenic differentiation of BMSCs.

The maintenance of adult bone mass is regulated by the state of the balance between osteoblasts and osteoclasts and changes in their life cycle caused by changes in apoptosis (95), which result in the destruction of at least 60% of osteoblasts (96). Therefore, the regulation of apoptosis in BMSCs, to attenuate bone metabolism disorders, is now being considered as an important means to alleviate osteoporosis. Although the exogenous injection of Apelin-13 significantly decreases serum deprivation-induced apoptosis, mitochondrial depolarization, cytochrome c release, caspase-3 activation, and reactive oxygen species production in a concentration-dependent manner, thereby increasing ERK1/2 phosphorylation and Akt protein expression, these protective effects can be effectively reversed by the PI3K inhibitor wortmannin or the ERK blocker UO126 (97). It has been suggested that Apelin-13 might mediate apoptosis by regulating a variety of signaling pathways. However, the *in vitro* injection of recombinant Apelin-13 failed to affect the protein expression of p-Akt, p-ERK, and p-JNK in human BMSCs (12); however, the researchers did not further explore these results, which are probably be attributed to different sources of BMSCs and different types of Apelin-13.

**Figure 3:**
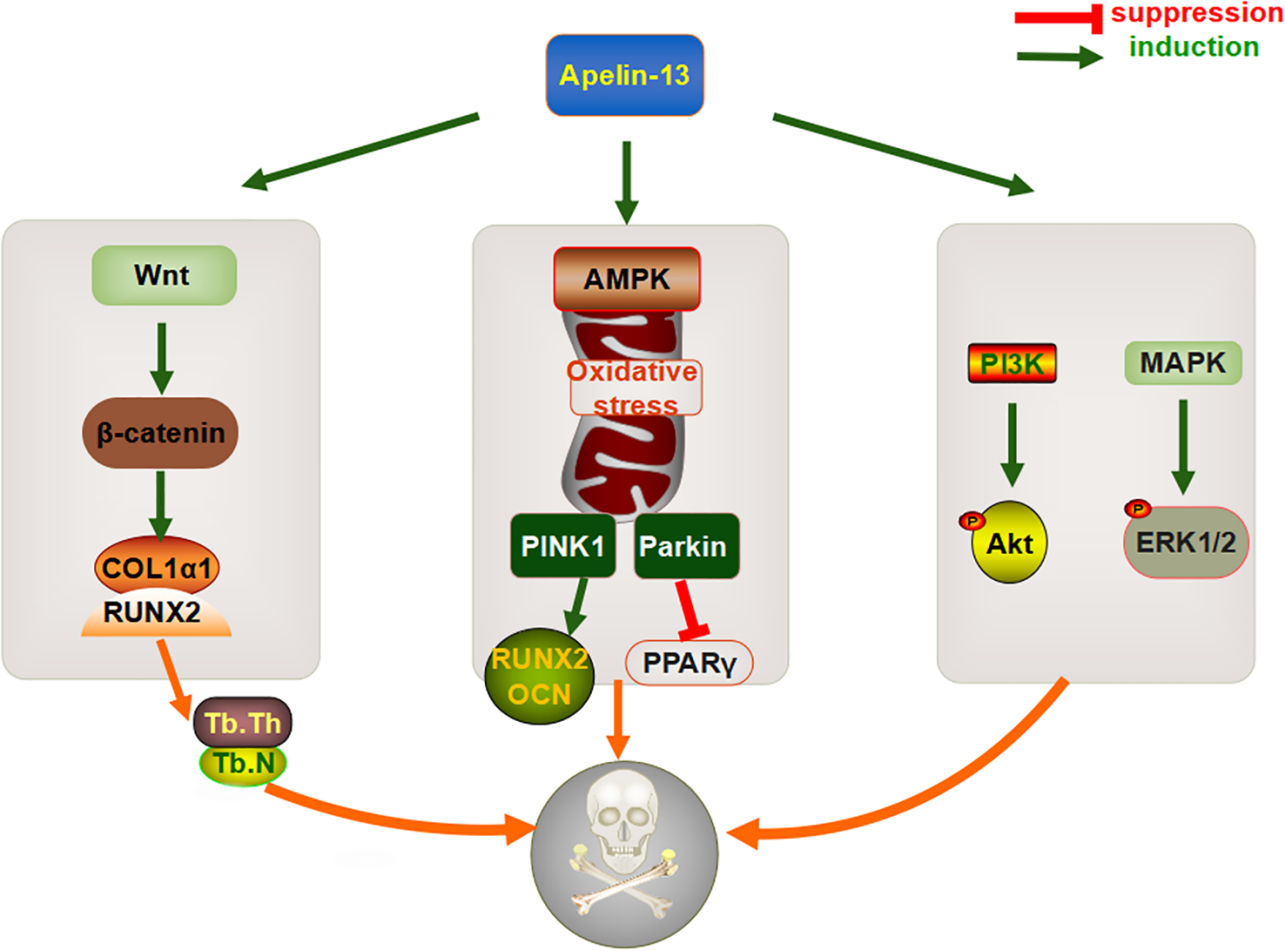
Mechanism through which Apelin-13 regulates bone metabolism.

In summary, Apelin-13 might affect osteogenic differentiation, based on effects on bone metabolism, directly via the Wnt/β-catenin pathway or indirectly via mitochondrial autophagy and apoptosis (**Figure 3**). However, its dual regulatory role in bone metabolism and related molecular mechanisms still require more systematic and in-depth research, especially with respect to osteoclasts, to provide a theoretical basis for the development of Apelin-related drugs.

## 4 Association between Apelin-13 and exercise

To further promote the utility of exercise for the prevention and control of metabolic diseases, it is necessary to study the molecular mechanisms associated with exercise-sensitive factors (98). Exercise can reduce or increase corresponding tissue-specific exercise factors, such as adiponectin (ADPN), irisin, and fibroblast growth factor 21 (FGF21) (99–101),, thereby attenuating a variety of metabolic diseases (102). One study found that peak oxygen consumption and serum Apelin-13 levels in obese men are increased significantly after undergoing resistance training, which involved 55 min of weight training, exercise at 3 days/week for 12 weeks, followed by a rest period of 4 weeks, and intermittent aerobic training, at 80–90% of the maximum heart rate × 4 min, with 3 min intervals between each session, at intervals of 65% of the maximum heart rate. After 4 weeks of training, the subjects maintained Apelin-13 levels, and neither their body fat rate nor their waist circumference returned to the pre-exercise state (17), suggesting that resistance training and intermittent aerobic training could directly affect Apelin-13 expression and promote its increase following exercise. The absence of an obvious difference in serum Apelin-13 levels between obese men and lean subjects at the beginning of the experiment compelled the authors to conclude that Apelin-13 expression in the body is not sufficiently related to the degree of obesity (17). Another study involved a maximum incremental load treadmill test, with a warm-up at 8 km/h for 2 min, followed by an increase to 10 km/h, with the slope maintained at 0% for the first 3 min, which was then increased by 1.5% per min, until the subject was unable to continue (volitional failure), at which time the test was ended. This experiment showed that although the serum level of Apelin-13 in some male professional soccer players was temporarily increased for 30 min after the exercise, in another group, this level decreased to a certain extent and showed individual differences (16). This result might have been related to the test status of each research subject. In addition, the rate at which Apelin-13 increased was found to be positively correlated with the maximum metabolic equivalent, relative maximum oxygen consumption, and maximum circulating power (16), indicating that Apelin-13 could be associated with sports potential, although this type of sports performance might also be due to the adaptation that occurs in professional athletes after long-term training. In conclusion, exercise can significantly upregulate Apelin-13 levels in the serum of male subjects, and these are closely associated with physical function, whole-body oxygen consumption, and cardiac pumping capacity. However, information regarding Apelin-13 levels in the circulation of women appears to be lacking, and the issue of gender dimorphism needs to be studied in relation to Apelin-13.

Most studies have indicated that Apelin-13 might regulate bodily activities via the central nervous system. The chronic intraventricular injection of Apelin-13 (1 μg/day; injection time of more than 24 h) significantly increases the nocturnal activities of female mice (36), suggesting that Apelin-13 might enhance the excitation of mouse motor neuron centers, thereby promoting increased activity. Although Ying et al. reported that the injection of Apelin-13 can enhance the discharge frequency and motor behavior of pale neurons and that unilateral injection can cause contralateral posture deflection in mice, thereby suggesting that Apelin-13 might directly participate in the regulation of limb movement in these animals (103), they did not elaborate on this. In terms of pathological conditions, Apelin-13 injection into the tail vein of male rats with cerebral ischemia was found to prevent NO depletion, thereby reducing neuronal death and the cerebral infarct volume and significantly improving sensorimotor balance defects (104).

In conclusion, different forms of exercise might upregulate the expression of Apelin-13 in serum. Under various physiological and pathological conditions, Apelin-13 could regulate motor centers, promote muscle contraction, and increase bodily activity. This effect implies that it plays a potential role in the neuromuscular system. Apelin-13 might also mediate the increase in energy expenditure during exercise, thereby improving and regulating metabolic diseases. In addition, cross-talk between Apelin-13 and exercise-associated factors, as well as the mechanisms underlying such crosstalk, remains unclear, indicating that the specific role of Apelin-13 in a variety of pathological states and the manifestation of exercise might need further evaluation.

## 5 Summary and outlook

In conclusion, Apelin-13, which is an adipokine, might play a heterogeneous role in the regulation of different pathological states, such as obesity, diabetes, osteoporosis, and other metabolic diseases. Although injecting Apelin-13 at a physiological level can cause metabolic disorders, under pathological conditions, it can also protect against metabolic diseases by regulating homeostasis via a variety of signaling pathways. Although the current study reviewed a variety of biological functions that involve Apelin-13, complete characterization of the specific mechanisms underlying the function of Apelin-13 has not been achieved. This also requires an in-depth exploration of the dynamic changes in the Apelin-13 regulatory network under different pathological states and the identification of key nodes. In addition, research on the regulatory effects of exercise on Apelin-13 is still in its formative stages. The regulatory effects of different types, intensities, and amounts of exercise on Apelin-13, which result in a protective effect on the body, remain unknown at present. Thus, an in-depth exploration of the multiple regulatory mechanisms associated with Apelin-13 and the development of related clinical therapeutic drugs will provide more new ideas and individualized targets for the prevention and treatment of metabolic diseases.

## Author contributions

RW: Writing – original draft. RH: Writing – review & editing. KX: Conceptualization, Investigation, Writing – review & editing. YC: Project administration, Supervision, Writing – review & editing. XY: Funding acquisition, Resources, Writing – review & editing.
